# Redesigning Nature: Ruthenium Flavonoid Complexes with Antitumour, Antimicrobial and Cardioprotective Activities

**DOI:** 10.3390/molecules26154544

**Published:** 2021-07-27

**Authors:** Nádia E. Santos, Susana Santos Braga

**Affiliations:** LAQV-REQUIMTE, Department of Chemistry, University of Aveiro, 3810-193 Aveiro, Portugal; nadiaasantos@ua.pt

**Keywords:** ruthenium, organometallic complexes, polypyridylic complexes, trithiacyclononane complexes, acqua complexes, cytotoxicity, antimicrobial action, cholesterol, anti-thrombotic action

## Abstract

Flavonoids are a class of natural polyphenolic compounds sharing a common 2-phenyl-3,4-dihydro-2*H*-1-benzopyran (flavan) backbone. Typically known for their antioxidant activity, flavonoids are also being investigated regarding antitumour and antimicrobial properties. In this review, we report on the complexation of both natural and synthetic flavonoids with ruthenium as a strategy to modulate the biological activity. The ruthenoflavonoid complexes are divided into three subclasses, according to their most prominent bioactivity: antitumour, antimicrobial, and protection of the cardiovascular system. Whenever possible the activity of the ruthenoflavonoids is compared with that of commercial drugs for a critical assessment of the feasibility of using them in future clinical applications.

## 1. Introduction

**Figure 1:**
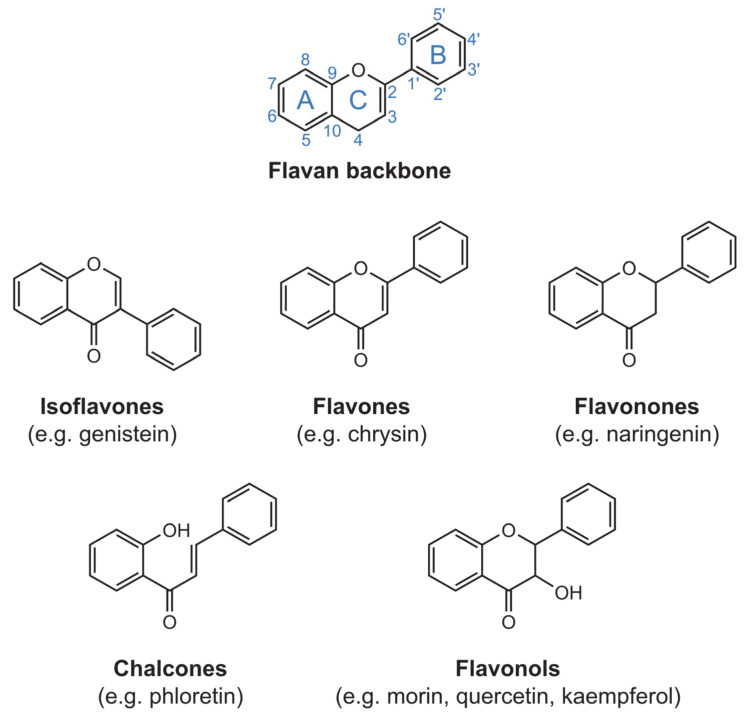
Structural representation of the flavan backbone, depicting the typical ring labeling scheme, and of the families of flavonoids of interest to the present review. Examples of compounds belonging to each family are given below the family name.

Flavonoids are polyphenolic compounds found in a wide variety of natural sources, from fruits and vegetables to tea leaves and cocoa. Flavonoids are well-known for their antioxidant activity, but recent studies have shown that these compounds have a broader range of biological properties, which include cytotoxic [1], chemopreventive [2], antimicrobial and even antithrombotic actions. The class of the flavonoids comprises various families of structurally related compounds. For the purpose of the present review, we highlight the ones that have reportedly formed complexes with ruthenium: flavones, isoflavones, flavonones, flavonols, and chalcones (Figure 1). These compounds all share the flavan structure as a common backbone.

Flavonoids are very safe compounds, with many already available from commercial sources for human intake as nutritional supplements. Examples range from plant extracts rich in flavonoids, such as ginkgo biloba extracts [3] and procyanidin blends [4], to pure compounds like fisetin [5], kaempferol [6], and quercetin [7,8], which is an FDA-approved supplement with the GRAS status (Generally Regarded as Safe) at doses up to 500 mg per serving [9]. Limitations in the use of flavonoids include poor bioavailaility from oral intake and quick metabolisation and elimination. With quercetin, for instance, only 20–30% of the oral dose is bioavailable [10]. A second but less expressive limitation to the ingestion of flavonoids is their possible interaction with a few other drugs. For quercetin, high doses (4.5 g daily) were reported to increase the bioavailability of the anti-histamine drug fexofenadine [11] and a lower dose (500 mg daily) increased the bioavailability of pravastatin, a cholesterol-regulating drug [12].

The association of flavonoids with ruthenium complexes via coordination of substituents at different positions is a strategy to fine-tune the electronic properties of the flavonoids, thus originating complexes with new electrochemical and biochemical activity. It may even help to reduce flavonoid-drug interactions because of the stabilisation brought about by complexation. Ruthenium complexes may also bring biological properties themselves. Having ruthenium as the metal centre for a complex brings several advantages for biological applications: (i) Ru complexes are very safe and well tolerated in vivo; (ii) they mimic iron, which allows them to bind to transferring inside the organism and thus be transported through the bloodstream without suffering from interactions and inactivation by other proteins; (iii) they allow fine-tuning reactivity by use of labile or kinetically inert ligands. The safety profile and biophysical properties of ruthenium complexes have been the target of various studies, which were developed since the 1960s and have contributed to consolidation of them as safe molecules to use in the medicinal field. An overview of the main findings is presented in the subsection below.

### Pharmacologic and Biophysical Properties of Model Ruthenium Complexes

Simple polypyridylic complexes such as [Ru(phen)_3_Cl_2_] (phen = 1,10-phenanthroline) or complexes with other heterocyclic ligands are known since the nineteen-fifties to have biocidal activity against bacteria, yeasts, and even viruses [13,14,15,16]. The mode of action of these complexes was often related with DNA intercalative properties [17]. Moreover, complexes such as [Ru(Me_4_phen)_3_]^2+^ (where Me_4_phen is a strongly lipophylic ligand, 3,4,7,8-tetramethylphenanthroline) have shown the ability to depolarise the bacterial membrane [18].

The biophysical properties and safety profile of the different salts of the complex cation [Ru(phen)_3_]^2+^ are well-known [14,15]. For *rac* [Ru(phen)_3_](ClO_4_)_2_, for instance, toxicity in mice from intraperitonial administration was >18.4 mg/Kg of body weight (meaning that the highest tested dose, 18.4 mg/Kg, did not cause lethal effect on the mice); oral toxicity was much lower, with values of 80 mg/kg having no effect on the mice, which was interpreted as the result of poor oral bioavailability [13] (probably due to low permeability). Biodistribution and fate studies of this complex (using a radiolabelled form, [Ru^106^(phen)_3_]^2+^, given intraperitoneally) have shown widespread distribution with high concentrations at the liver, kidney, diaphragm, pancreas, and spleen, and lower concentrations in the lungs, intestine, suprarenal glands, testis, heart, skeletal muscles, eye, and skin; no traces were found in the brain tissue. Elimination through the urine led to 97% excretion of the complex within 24 h [19].

A large variety of organometallic ruthenium arene complexes with antitumor [20,21] and anti-parasitary [22,23] activities are reported. Within antitumorals, one of most thoroughly studied complexes is [RuCl(*p*-cymene)(1,3,5-triaza-7-phosphaadamantane)], commonly abbreviated as RAPTA-C [24]. RAPTA-C is safe to administer intravenously to mice at a dose of 400 mg/kg, both when given all at once and when divided in 4 × 100 mg/kg (given every two days) [25]. Pharmacokinetic studies of RAPTA-C in mice from a single intravenous dose of 200 mg/kg showed distribution to the main organs: liver, kidney, spleen, and lung (we note that its presence in other organs and tissues was not investigated) and a quick elimination via the kidneys, with a total half-life (*T*_1/2_) of c.a. 11.5 h [25]. Retention of the complex in the different organs was also measured by assessing the different times of half-life in each organ. Accumulation is more expressive in the spleen, with *T*_1/2_ = 61.2 h, followed by the liver, with *T*_1/2_ = 32.4 h and the kidney and lung (*T*_1/2_ of 14.2 and 15.1 h, respectively).

## 2. Ruthenium Flavonoid Complexes with Cytotoxic Activity In Vitro

### 2.1. Complexes Obtained from [Ru(DMSO)_4_Cl_2_]

**Figure 2:**
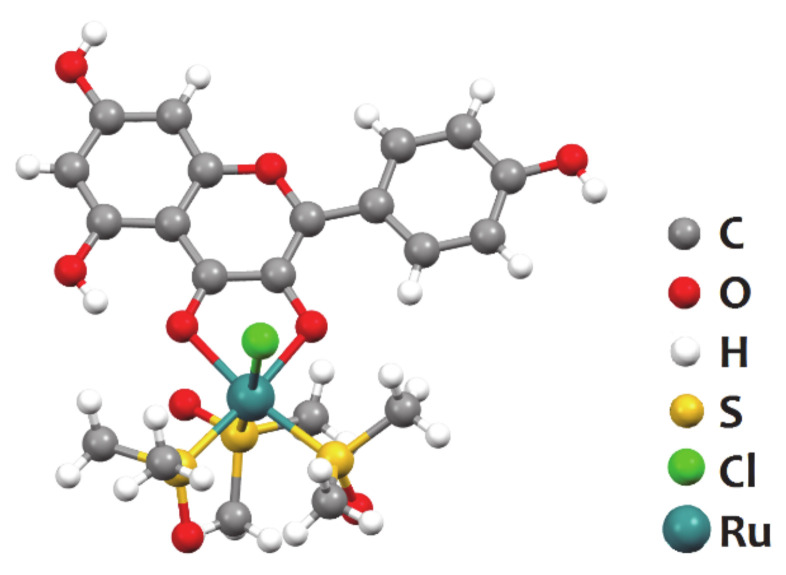
Structure of [Ru(DMSO)_3_(kaempferol)Cl], represented using the ball-and-stick style. Redrawn from the atomic coordinates available at the CCDC (refcode XETLEK) using the software Mercury 3.7.

A few of the simplest structures of ruthenium flavonoids complexes are obtained from reaction with the inorganic ruthenium precursor [Ru(DMSO)_4_Cl_2_] (DMSO stands for dimethylsulfoxide), which can itself be prepared by merely refluxing the commercial RuCl_3_ salt in DMSO for 24 h. There are two types of these complexes described in the literature, some having only one flavonoid and others having two flavonoid ligands in their coordination sphere. Thangavel et al. reported the complex [Ru(DMSO)_3_(kaempferol)Cl] (Figure 2), which featured an IC_50_ of c.a. 10 μM against the lung cancer A549 cell line while having minimal toxicity on healthy HDFa cells (human dermal fibroblasts); pure kaempferol was also tested for comparison, having shown an IC_50_ around 20 μM [26].

**Figure 3:**
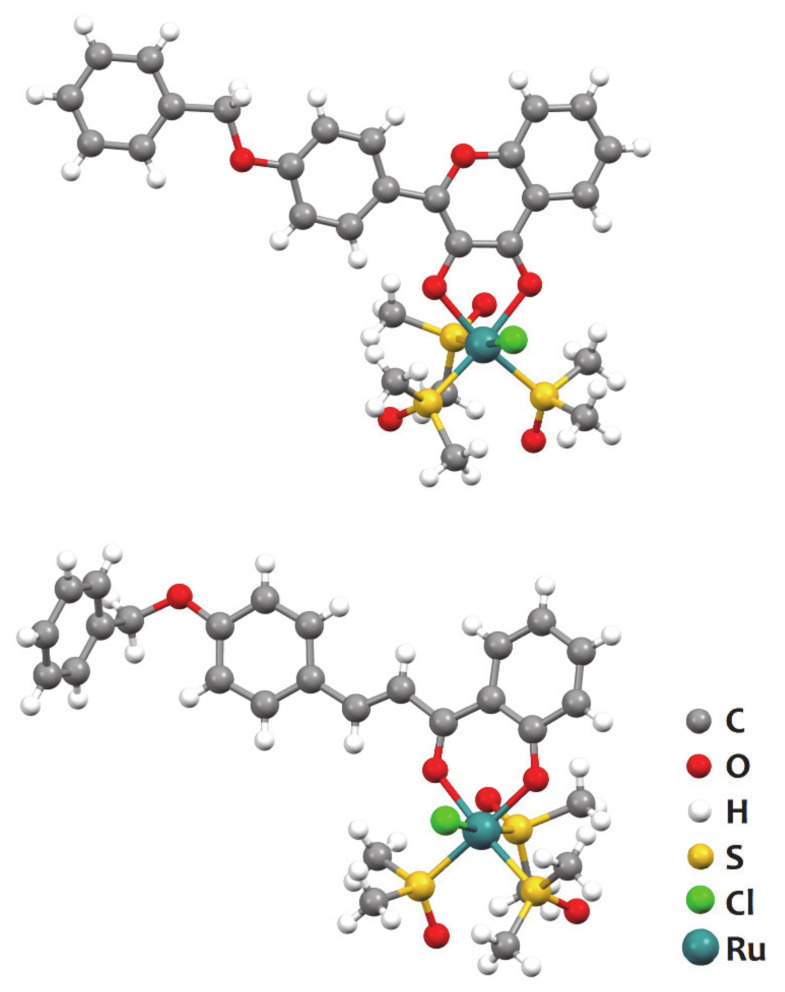
Structure of [Ru(DMSO)_3_(FLVD)Cl], where FLVD is 2-(4-benzyloxy-phenyl)-3hydroxychromen-4-one) (top) and [Ru(DMSO)_3_(CHRD)Cl], where CHRD is 3-(4-benzyloxyphenyl)-1-(2-hydroxylphenyl)-prop-2-en-1-one (bottom), represented using the ball-and-stick style. Redrawn from the atomic coordinates available at the CCDC (refcodes TUSTAD and TUSTEH) using the software Mercury 3.7.

Ru(II) complexes with a single flavonoid derivative as the ligand were also reported by Prajapati et al. (Figure 3). The organic ligands were not natural flavonoids but rather chemically modified derivatives of a chalcone and a flavone: 3-(4-benzyloxyphenyl)-1-(2-hydroxylphenyl)-prop-2-en-1-one and 2-(4-benzyloxy- phenyl)-3-hydroxy-chromen-4-one [27]. Both complexes exhibited excellent activity against Dalton lymphoma cells, with IC_50_ values of 0.32 and 0.82 μM at 48 h of incubation, while for the pure chalcone and flavone the IC_50_ values were 1.34 and > 5 μM.

**Figure 4:**
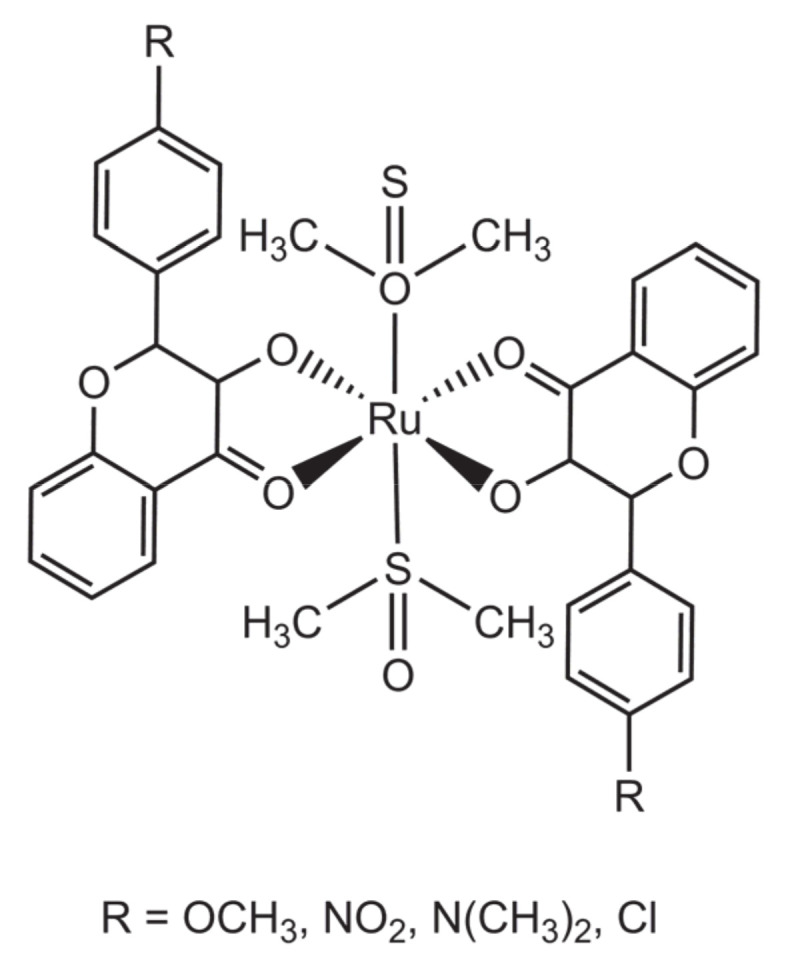
Structural representation of set of four ruthenium dimethylsulfoxide complexes bearing two flavonol-derived ligands.

A family of four complexes, each with two flavonol ligands, was reported by Singh et al. [28]. The complexes, represented in the Figure 4, differ only in the substituent group on the B ring of flavonol. The complexes were tested regarding their anti-proliferative activity against breast cancer MCF-7 cells, having displayed IC_50_ values between 16.0 and 36.2 μM. These values are within the same range as those obtained with the pure flavonol ligands, which were between 17.2 and 38.4 μM. Two possible interpretations can be outlined for these results. The first is that the presence of two flavonoid ligands in the coordination sphere may create some degree of steric hindrance that would diminish the accessibility of the labile ligands (in this case, DMSO) and, thus, their ability to interact with biomolecular targets; this would make the reactivity of these complexes become more similar to that of the pure ligands. The second interpretation is that the results reflect the sensitivity of the cells of this particular breast cancer line (MCF-7) to chemotherapeutic agents: MCF-7 cells are an oestrogen receptor positive line, being thus highly responsive to molecules that mimic oestrogen, such as flavonoids, and not so much to the action of the ruthenium metal.

### 2.2. Ruthenium Acqua Complexes

**Figure 5:**
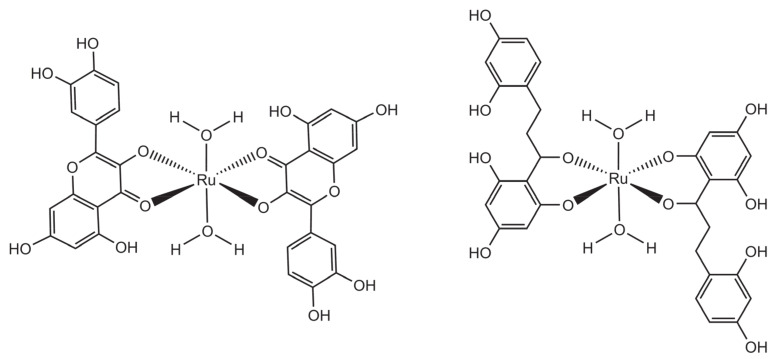
Structural representation of the complexes [Ru(quercetin)_2_(H_2_O)_2_] (left) and [Ru(phloretin)_2_(H_2_O)_2_] (right).

Recently, two ruthenium diflavonoid complexes were reported, starting from the RuCl_3_ salt and methanol solutions of quercetin [29] and phloretin [30]. Interestingly, both products precipitated as diacqua complexes (Figure 5), as verified by their complete spectroscopic characterisation (^1^H NMR, UV-Vis, FT-IR and mass spectrometry).

The biological activity of the two complexes was evaluated both in vitro and in vivo. For [Ru(phloretin)_2_(H_2_O)_2_], incubation at a concentration of 100 μM, for 24 h, with human colon adenocarcinoma HT-29 cells caused cell viability to lower to 59.6%; the complex was further shown to induce apoptosis on this cell line [30]. Studies with mice investigated both the safety and the anti-tumour efficacy of [Ru(phloretin)_2_(H_2_O)_2_]. The complex is relatively well tolerated, with an LD_50_ of 400 mg/kg and some hepatotoxicity (increased transaminases and alkaline phosphatase) at a dose of 300 mg/kg. Moreover, the complex was shown to be effective in both chemoprevention and chemotherapy: a dose of 200 mg/kg strongly reduced colon aberrant crypt formation in mice challenged with a carcinogenic agent; in mice that were previously treated with a carcinogenic agent to induce colon cancer, treatment with 200 mg/kg of [Ru(phloretin)_2_(H_2_O)_2_] led the hyperplasic lesions to resume their normal histological aspect, thus hinting at full tumour recovery.

The complex [Ru(quercetin)_2_(H_2_O)_2_] was also tested against the HT-29 cell line, with a test concentration 100 μM having lowered cell viability to 45.2%; The complex was further shown to interact with DNA at the major groove and to induce apoptosis of HT-29 cells with associated chromatin condensation [29]. Acute and sub-acute toxicity evaluation was conducted on mice, having an LD_50_ of 600 mg/kg and a quite good safety, with no deaths occurring in animals treated with doses of 400 mg/kg or lower. The chemopreventive and chemotherapeutic in vivo activity of [Ru(quercetin)_2_(H_2_O)_2_] was similar to that of its phloretin counterpart, with 200 mg/kg preventing aberrant colon crypt formation in challenged mice and reducing tumour cell proliferation. Investigation into the mode of action of [Ru(quercetin)_2_(H_2_O)_2_] showed its antitumour activity to occur via p53-mediated apoptosis.

### 2.3. Ruthenium Polypyridyl Complexes

**Figure 6:**
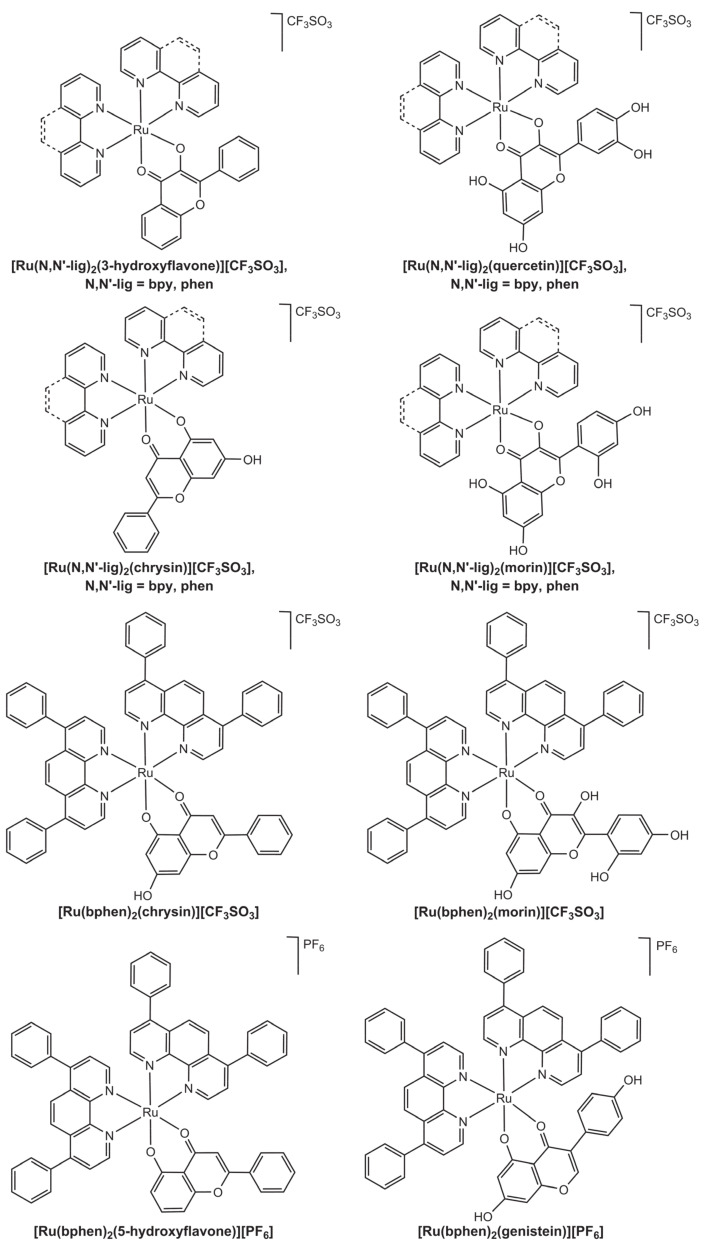
Structural representation of ruthenium polypyridyl complexes of the type [Ru(N,N’-lig)_2_(flv)]^+^. Two families of complexes are reported, one having N,N-lig = 2,2′ bipyridine (bpy) or 1,10-phenanthroline (phen) and flv = 3-hydroxyflavone, quercetin, chrysin, or morin [31] and another family having N,N’-lig = *bis*-bathophenanthroline (bphen) and flv = 5-hydroxyflavone, genistein, chrysin, and morin [32]. Note that the unusual coordination at the 4,5-*O*,*O* site of the morin complex observed in the second family was achieved by means of a taylor-made synthesis that resorted to protective groups. Morin typically coordinates via the 3,4-*O*,*O* position, as observed in the corresponding complexes of the first family.

A family of cationic ruthenium polypyridyl complexes with quercetin, morin, chrysin and 3-hydroxyflavone and having 2,2′-bipyridine and 1,10-phenanthroline as co-ligands was reported (Figure 6) [31]. The biological activity of the complexes was evaluated in four tumour cell lines: cervical carcinoma (HeLa line), colorectal adenocarcinoma (SW620 line), hepatic adenocarcinoma (HepG2 line) and breast cancer (MCF-7 line), with IC_50_ values of selected compounds being presented in the Table 1. We note that many of the results are inconclusive because they reflect aberrant data. For instance, looking at the IC_50_ (expressed as mean ± SD) reported for SW260 cells incubated with [Ru(bpy)_2_(3-hydroxyflavone)][CF_3_SO_3_], one finds the value of 8.2 ± 46.4 μM, which is indicative of an abnormal data distribution (i.e., cell growth was, most likely, not reproducible between the assays). Nevertheless, it is still possible to note that [Ru(bpy)_2_(3-hydroxyflavone)][CF_3_SO_3_] had very promising antiproliferative activity against most of the tested cell lines, with IC_50_ values in the low micromolar range, whereas pure 3-hydroxyflavone did not present such strong activity (Table 1). In the case of the flavonoids quercetin, morin and their corresponding ruthenium complexes with bpy and phen, no activity was reported against the tested tumour cell lines (IC_50_ > 100 μM). Finally, results for chrysin and its corresponding bpy and phen complexes were also inconclusive due to abnormal data distribution.

A second family of cationic ruthenium polypyridyl complexes with flavonoids was recently developed and tested regarding the antiproliferative action on a collection of cancer cell lines [32]. The complexes had the general formula [Ru(bphen)_2_(flv)]^+^, where bphen is 4,7-diphenyl-1,10-phenanthroline (bathophenanthroline) and flv is 5-hydroxyflavone, genistein, chrysin, or morin. Two of the complexes readily precipitated as triflate salts (triflate was present in the reaction media), while the other two required addition of phosphate hexafluoride to precipitate by forming the corresponding salt (Figure 6).

ESI-MS and NMR studies (both 1D and 2D) helped confirm the structure and coordination mode for each complex. NMR was also employed to monitor the stability of the complex [Ru(bphen)_2_(morin)][CF_3_SO_3_] with results showing that, even though it was purposely obtained with 4,5-*O*,*O* coordination for morin (by blocking the other available coordination sites of morin with protecting groups), it tended to slowly revert to its more stable 3,4-*O*,*O*-coordinated isomer, with 25% conversion observed after 5 days in solution. The biological activity of the complexes was tested on four tumour cell lines, namely two breast cancer lines, MDA-MB-435S and MCF-7, one pharynx carcinoma FaDU cell line, and one glioblastoma U87 cell line, and on two immortalised cell lines: retinal pigmented epithelium RPE-1 cell line, and embryonic kidney HEK 293 line. In tandem, two known antitumor drugs (cisplatin and doxorubicin) were used as positive controls [32]. Results, compiled in Table 2, show that three of the pure flavonoids, 5-hydroxyflavone (5OHFlv), genistein (gen) and morin (mor) were inactive against all the tested tumour cell lines, while chrysin had some cytotoxic activity, which was more expressive against the immortalised kidney HEK 293 cell line and the MCF-7 breast cancer cell line. Regarding the complexes, [Ru(bphen)_2_(morin)][CF_3_SO_3_] did not display cytotoxic activity, with the other three complexes had some cytotoxic action, the potency depending on the tested cell lines. Highlight must be given to [Ru(bphen)_2_(gen)][PF_6_], which was the most potent complex of this family, matching the activity of doxorubicin and, on some of the cell lines, being more potent than this model drug. Mechanistic studies into the mode of action of [Ru(bphen)_2_(gen)][PF_6_] showed that it is able to enter some cells (e.g., MDA-MB-435S) by passive diffusion and that higher accumulation inside these cells is responsible for the stronger cytotoxic effect observed. Comparing intracellular accumulation of [Ru(bphen)_2_(gen)][PF_6_] inside the two breast cancer cells, MDA-MB-435S and MCF-7, higher concentration was observed inside the first cell line, in direct correlation with the lower IC_50_. The complex was further shown to inhibit mitochondrial respiration, strongly lowering the cellular production of ATP.

### 2.4. Ruthenium Trithiacyclononane Complexes

**Figure 7:**
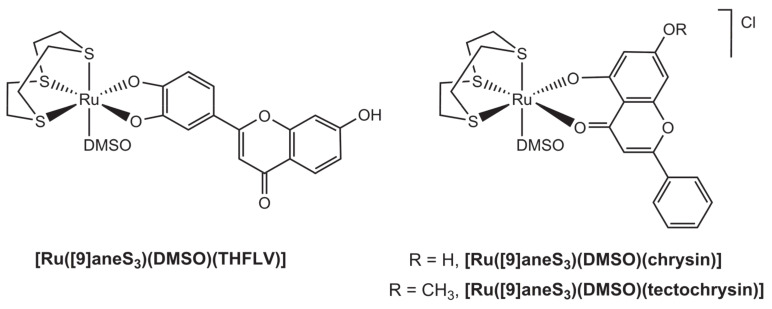
Structural representation of the neutral complex [Ru([9]aneS_3_)(DMSO)(THFLV)], with [9]aneS_3_ = trithiacyclononane and THFLV = (*bis*-deprotonated) 7,3′,4′-trihydroxyflavone (left) and the cationic complexes [Ru([9]aneS_3_)(DMSO)(chrysin)]Cl and [Ru([9]aneS_3_)(DMSO)(tectochrysin)]Cl (right).

Three ruthenium trithiacyclononane complexes, having the ligands 7,3′,4′-trihydroxyflavone (THFLV), chrysin (or 5,7-dihydoxyflavone) and tectochrysin (or 5-hydroxy-7-methoxyflavone), were recently reported (Figure 7) [33]. Interestingly, coordination with 7,3′,4′-trihydroxyflavone occurred at the catechol moiety of the B-ring to form a five-membered coordination ring that is the first reported example of ruthenium-catechol coordination in polyaromatic phenolic ligands; note also that the flavone suffered loss of two protons and coordinated as a dianionic ligand to form a neutral complex. In turn, chrysin and tectochrysin coordinated via the 4 and 5 positions, as typically observed in ruthenium complexation with flavonoids; these ligands suffered only one deprotonation, having thus formed monocationic complexes with the Ru(II) scaffold.

The complexes and their corresponding flavone ligands (for comparison) were tested against the prostate cancer PC-3 line, the osteosarcoma MG-63 cell line, and two breast cancer lines, MCF-7 and MDA-MB-231. The flavones displayed moderate activity, with IC_50_ values within the range of 16–41 μM for 7,3′,4′-trihydroxyflavone and chrysin (against all tested cell lines) and tectochrysin having shown a lower activity, its lowest IC_50_ having been c.a. 33 μM against the prostate cancer line (PC3) and its highest IC_50_ having been 113.5 μM against the estrogen-dependent breast cancer line (MCF-7). Antitumor activity was, however, lost upon complexation with the ruthenium trithiacyclononane scaffold: the complexes with chrysin and tectochrysin displayed IC_50_ > 200 and, in the case of [Ru(trithiacyclononane)(THFLV)(DMSO)]Cl, the low solubility in water (<18.5 μM) rendered it unsuitable for biological applications.

### 2.5. Ruthenium p-cymene Complexes

**Figure 8:**
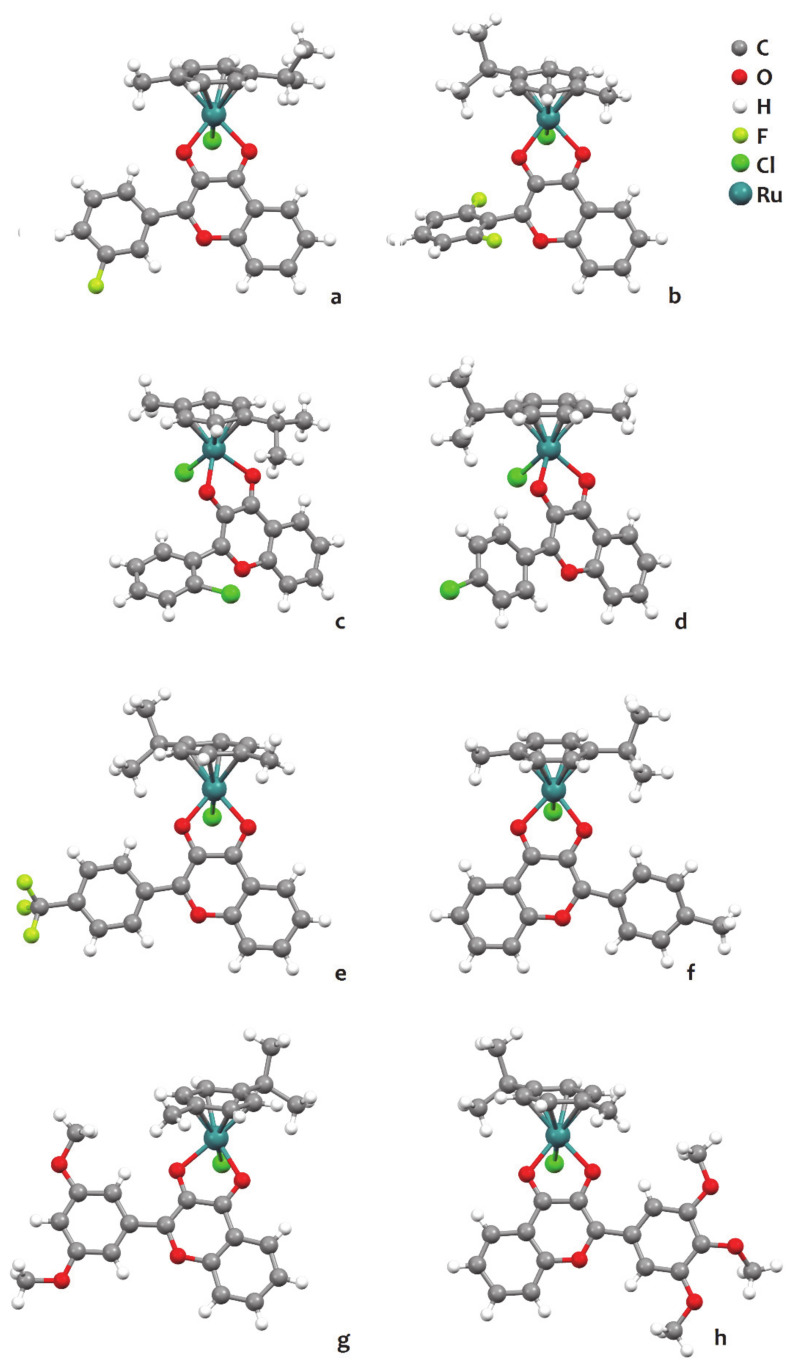
Structures of ruthenium *p*-cymene (*p*-cym) complexes with flavonol (3-hydroxyflavone) derivatives: (**a**) [Ru(*p*-cym)(3′-fluoroflavonol)Cl] (CCDC refcode VEMVAM); (**b**) [Ru(*p*-cym)(2′,5′-difluoroflavonol)Cl] (ODELER); (**c**) [Ru(*p*-cym)(2′-chloroflavonol)Cl] (VEMTOY); (**d**) [Ru(*p*-cym)(4′-chloroflavonol)Cl] (VEMTUE); (**e**) [Ru(*p*-cym)(4′-trifluoromethylflavonol)Cl] (ODELIV); (**f**) [Ru(*p*-cym)(4′-methylflavonol)Cl] (SARBIY); (**g**) [Ru(*p*-cym)(3′,5′-dimethoxyflavonol)Cl] (ODEKUG), and (**h**) [Ru(*p*-cym)(3′,4′,5′-trimethoxyflavonol)Cl] (ODELAN), all represented using the ball-and-stick style. Redrawn from the atomic coordinates available at the CCDC using the software Mercury 3.7, omitting co-crystalisation solvent molecules for clarity.

A family of organometallic ruthenium *p*-cymene (*p*-cym) complexes having flavonol derivatives with methyl or halogen substituents on the B ring was developed and tested on a small collection of cancer cell lines [34,35,36]. The crystal structures of various compounds of this family were collected, being shown in Figure 8. Overall, the complexes share a geometry deemed as “piano-stool”, in which the arene group (*p*-cym in the present case) forms the seat of the stool and the other ligands (chloride and flavone) protrude in three directions away from the ruthenium, at the center of the molecule, to form the legs of the stool.

Cytotoxicity tests were conducted on a small collection of human tumour cell lines which include the human ovarian CH1 cancer, the SW480 colon adenocarcinoma line, the 5637 urinary bladder grade II carcinoma line, the DAN-G pancreatic adenocarcinoma line, and two lung cancer lines, the A549 non-small cell lung line and the LCLC-103H large cell lung line. Results, compiled in Table 3, show that most of the complexes exhibited superior or, at least, identical activity to that of the pure flavonoids.

The most potent complexes were the ones bearing a halogen at the *meta* or *para* position of the B ring of flavonol. The ability to inhibit topoisomerase IIα was measured for a few of these complexes, having been found to correlate well with the in vitro anticancer activity data: the complexes exhibiting the lowest IC_50_ values were also the most potent topoisomerase IIα inhibitors [34]. Moreover, coordination of the flavonols to the ruthenium *p*-cym scaffold increased the topoisomerase-inhibiting activity (in comparison with the pure flavonols). This was postulated to result from the ability of the complexes to form bonds with the DNA base guanine [35]. Regarding the complexes with methylated [34] or methoxylated flavonol [36], activity was somewhat lower, but still higher than that of the complexes with 2′-halogenated flavonol [35], the less potent ones in this family (Table 3).

**Figure 9:**
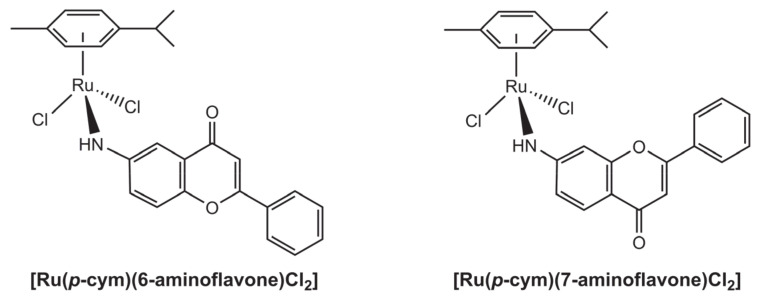
Structural representation of two ruthenium *p*-cymene complexes bearing aminoflavone ligands.

Two ruthenium *p*-cymene complexes with 6- and 7-aminoflavones were developed and tested against melanoma and leukaemia cell lines [37]. The aminoflavone in these complexes is coordinated monodentately by the nitrogen atom, as represented in the Figure 9. Regarding the activity, both the pure aminoflavones and the complexes were inactive against the k562 leukaemia cell line, even when incubated at high concentration, 100 μM. The same concentration of [Ru(*p*-cym)(6-aminoflavone)Cl_2_] lowered the viability of A375 melanoma cells to c.a. 83%, a poorer result than that observed with the pure ligand, 6-aminoflavone (c.a. 64% viability); both 7-aminoflavone and its Ru complex were inactive against this melanoma line. With primary melanoma cells (lines DMBC11 and DMBC12), results were more promising, the complexes having caused viability to drop to 50–55%. Their activities were, however, always lower than those of the corresponding pure aminoflavones.

## 3. Antimicrobial Ruthenium Flavonoid Complexes

Metal-based compounds are long known to have biocidal properties. Inorganic copper compounds are traditionally used in crop science, particularly the colloidal mixture of blue copper hydroxide adsorbed with copper sulphate (Bordeaux mixture, obtained from copper sulfate pentahydrate mixed with lime) [38]. Silver compounds, used since ancient times in disinfection, are now having resurgence in medicinal and personal care products, with higher incidence in Asian countries [39]. Biocidal ruthenium complexes are known for several decades [40,41], with research focusing more intensively on the ones having polypyridyl ligands. Antimicrobial ruthenium complexes with flavonoids are still rare, with only three reports available in the literature [31,42,43]. Nevertheless, antimicrobial ruthenium flavonoids have the advantage of being adaptable to varied applications, as demonstrated by two relevant examples: (i) an exploratory family of antimycobacterium agents [42] and (ii) ruthenoflavonoid complexes suitable as agrochemical agents for the management of a threatening crop disease [43].

### 3.1. Ruthenium Polypyrydil Complexes for Antimicrobial Action in Human Medicine

The first family comprises cationic ruthenium complexes of 2,2′-bipyridine or 1,10-phenanthroline and the flavonoids quercetin, morin, chrysin and 3-hydroxyflavone [31]. These compounds were previously mentioned in the present review regarding their antitumour activity (see structures in Figure 6) [31]. They were studied regarding interaction with active proteins that can help with their transport inside the organism as well as with absorption, bioavailability and activity. Albumins being the most abundant proteins in plasma, commonly used to study this type of interactions, the authors selected bovine serum albumin (BSA), a model protein structurally similar to human serum albumin. Complexes with polyphenolic flavonoids, i.e., those with quercetin and morin (see Figure 6), were found to bind more strongly to BSA, indicating that the presence of free hydroxyl groups is quite relevant for the interaction, perhaps due to possibility of forming additional hydrogen bonds with BSA. Moreover, morin complexes bound more strongly to BSA when compared to their quercetin counterparts, suggesting the importance of the hydroxylation pattern of the flavonoid ring B. The antimicrobial activity of the ruthenium (II) flavonoid complexes was tested on several Gram-positive bacteria strains such as: *Staphylococcus aureus* ATCC 25923, *Enterococcus faecalis* ATCC 19433, *Streptococcus* beta-hemolytic group A, Methicillin-resistant *Staphylococcus aureus* (MRSA), on some Gram-negative bacteria strains such as: *Klebsiella pneumoniae* ATCC 1705, *Acinetobacter baumannii* ATCC-BAA 747, *Pseudomonas aeruginosa*, *Escherichia coli*, and on the unicellular fungus *Candida albicans.* The results, compiled into Table 4, show that the complexes [Ru(bpy)_2_(chrysin)Cl] and [Ru(bpy)_2_(3-hydroxyflavone)Cl] were active against Gram-positive bacteria, while the corresponding ligands were inactive; notably, the activity of [Ru(bpy)_2_(3-hydroxyflavone)Cl] against MRSA surpassed that of the reference antibiotic, gentamicin. [Ru(bpy)_2_(3-hydroxyflavone)Cl] was also more active against *C. albicans* than pure 3-hydroxyflavone, while activity toward the Gram-negative *A. baumannii* was identical for the complex and the flavone. These results show that the presence of a metal centre can contribute to afford antimicrobial activity to flavonoids that are inactive on their own. Nevertheless, this synergy is not always observed as, interestingly, the opposite effect occurred in the case of quercetin complexation: while free quercetin was moderately active against *S. aureus*, *E. faecalis* and MRSA*,* its corresponding ruthenium (II) complexes displayed no antimicrobial activity [31].

Another relevant note is that the complexes were, in the large majority, inactive against Gram-negative bacteria [31]. This was expected as similar results had been previously observed with antimicrobial Ru(II) complexes comprising Schiff bases as ligands [44,45]. The higher resistance of Gram-negative bacteria is associated with the presence, in these microorganisms, of an additional lipopolysaccharide layer along the outer membrane, consequently thickening the cell wall and affecting the diffusion-controlled transport of these antimicrobial agents.

**Figure 10:**
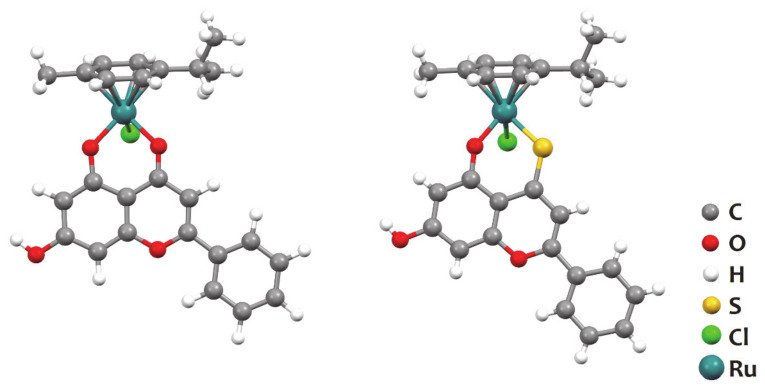
Structural representation of a family of dicationic Ru(II) complexes with the ligands 1,10-phenanthroline and hydrazine-flavone: [Ru(3-OH-4-hydrazine-4′-R-flavone)(phen)_2_]PF_6_.

A family of ruthenium (II) 1,10-phenanthroline complexes with 3-hydroxy-4-hydrazine-4′-R-flavones has been recently developed for antimycobacterial activity [42]. The complexes have the general formula [Ru(phen)_2_(3-OH-4-hydrazine-4′-R-flavone)], with R = OCH_3_, NO_2_, N(CH_3_)_2_, Cl, or OCH_2_Ph (Figure 10).

The growth-inhibitory activity of the complexes was studied on *Mycobacterium smegmatis*, a microorganism commonly used as a model for *M. tuberculosis* because it shares the same peculiar cell wall structure of *M. tuberculosis*. The complexes with highest activity were those with R = OCH_3_ and Cl, both with a minimum inhibitory concentration (MIC) value of 6.25 μM and a CFU (colony forming unit) reduction greater than 99% at MIC. These two complexes were further shown to bind to the DNA of *M. smegmatis* by two assays: direct titration monitored by UV-Vis spectroscopy and displacement of ethidium bromide (EtBr) from DNA. ETBr-DNA adducts are fluorescent due to the intercalation of EtBr into DNA. Loss of fluorescence results from changes in the intercalation of EtBr. This phenomenon was observed as the gradual decrease in fluorescence intensity upon adding increasing concentrations of the Ru(II) complexes, with no fluorescence being visible at concentrations of 7.5 μM or higher [42].

### 3.2. Ruthenium Polypyrydil Complexes against Crop-Damaging Bacteria

*Xylella fastidiosa* is a gram-negative bacterium that develops in the xylem of varied plants, from grapevines to orchard trees such as citrus, almond and olive trees. It causes severe damage to the plants, losses in productivity and, in extreme cases, the death of the plant. Treatment usually involves removal and destruction of affected plant segments and through the control of the mosquitoes that act as its vectors [46]. 

**Figure 11:**
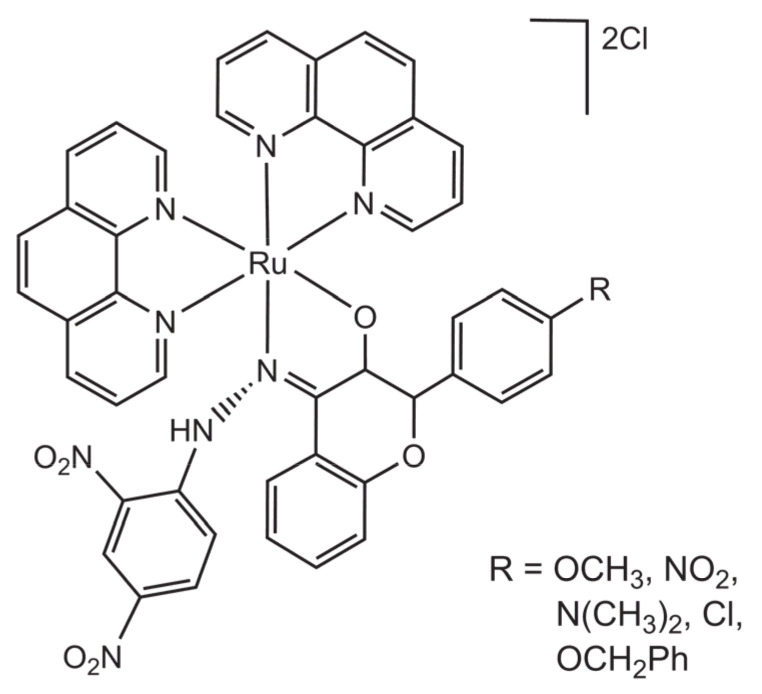
Structural representation of the cationic complex [Ru(naringenin)(phen)_2_]PF_6_.

Based on the ability of flavonols to disrupt biofilm formation, a common feature in *X. fastidiosa* infestation, a ruthenium 1,10-phenanthroline complex with naringenin as the active ligand was developed [43]. The complex has the general formula [Ru(naringenin)(phen)_2_]PF_6_, as depicted in Figure 11.

The antimicrobial activity of [Ru(naringenin)(phen)_2_]PF_6_ was tested both in vitro and in vivo. In vitro tests used the microdilution method to determine the MIC values of the complex and pure naringenin against *X. fastidiosa*, with streptomycin as the positive control. The complex [Ru(naringenin)(phen)_2_]PF_6_ had a remarkably low MIC of 0.19 μM, while naringenin had a MIC of 7.3 μM. Further studies were conducted in vivo in *Camellia sinensis* plants (grafted on *C. limonia* cv. Pêra) that were previously infected with *X. fastidiosa* via mechanical needle inoculation. The plants were then treated by injection of a solution (5 mL, at 5 mg·mL^−1^ concentration) of either naringenin or [Ru(naringenin)(phen)_2_]PF_6_. Positive and negative control groups of plants were also tested, with a solution of Cu(SO)_4_ for the positive control and water for the negative control. The amount of *X. fastidiosa* in the plants was evaluated after two months by qPCR. The results showed that plants treated with [Ru(naringenin)(phen)_2_]PF_6_ had a load of only 0.12% bacteria after the treatment, similarly to those treated Cu(SO)_4_; plants treated with naringenin had around 15.93% of the initial infection load [43]. These results are quite promising for two main reasons: (i) a 25 mg dose of [Ru(naringenin)(phen)_2_]PF_6_ is roughly equivalent to 0.28 μmol while the same mass dose of Cu(SO_4_) is equivalent to c.a. 15 μmol, which allows concluding that the Ru(II) complex is much more potent than the copper salt; (ii) development of bacterial resistance to copper sulphate is frequent, with the Ru(II) complex posing a suitable alternative to treat the disease in the case of copper-resistant strains.

## 4. Ruthenium Flavonoids Complexes in the Prevention and Treatment of Cardiovascular Diseases

Ruthenium arene complexes, in particular those having the *p*-cymene ligands, have been long explored for their antitumour properties, with some examples having been herein previously presented (in the Section 2.5). Nevertheless, the broad range of properties of flavonoids allows designing ruthenium complexes versed for other therapeutic applications, such as the regulation of cardiovascular diseases. Reported examples include the prevention of blood vessel blockage by a complex with cholesterol-lowering action as well as two complexes with anti-thrombotic properties.

### 4.1. A Ruthenium p-cymene Quercetin Complex for Cholesterol Regulation

**Figure 12:**
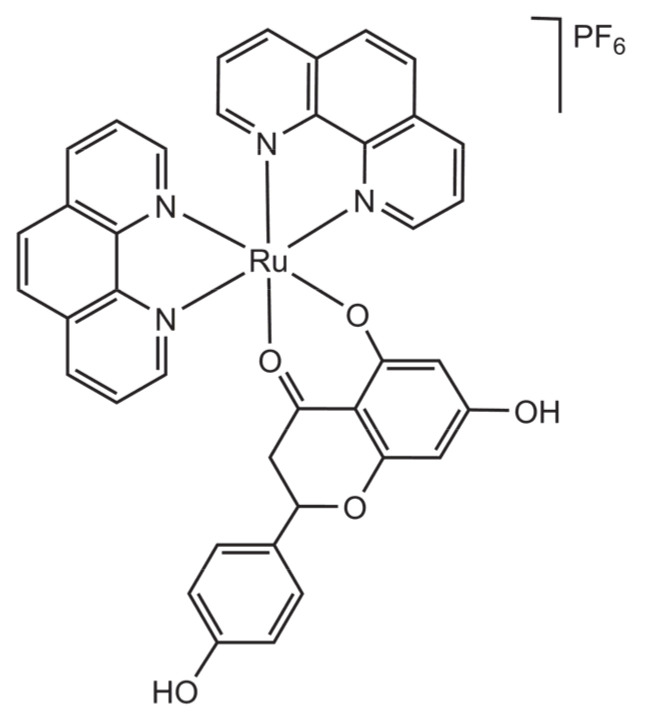
Structural representation of the complex [Ru(*p*-cym)(quercetin)Cl].

A ruthenium *p*-cymene complex with quercetin was recently reported (Figure 12). The complex was shown to bind to 3-hydroxy-3-methyl-glutaryl-CoA reductase (HMGR) both in silico and in vitro and to reduce the activity of this endoplasmic, reticulum-bound enzyme that regulates the early stages of cholesterol biosynthesis [47]. Further tests on cultured liver cells (HepG2 cell line) showed dose-dependent diminution of cytoplasmic cholesterol. Noteworthy, the complex [Ru(*p*-cym)(quercetin)Cl] had an activity significantly higher than pure quercetin and comparable to those observed for two model drugs, pravastatin and simvastatin. Moreover, the complex was very well tolerated by these cells, with lower cytotoxicity values than those of simvastatin itself.

### 4.2. Ruthenium p-cymene Complexes with Chrysin and Thiochrysin as Antithrombotic Agents

**Figure 13:**
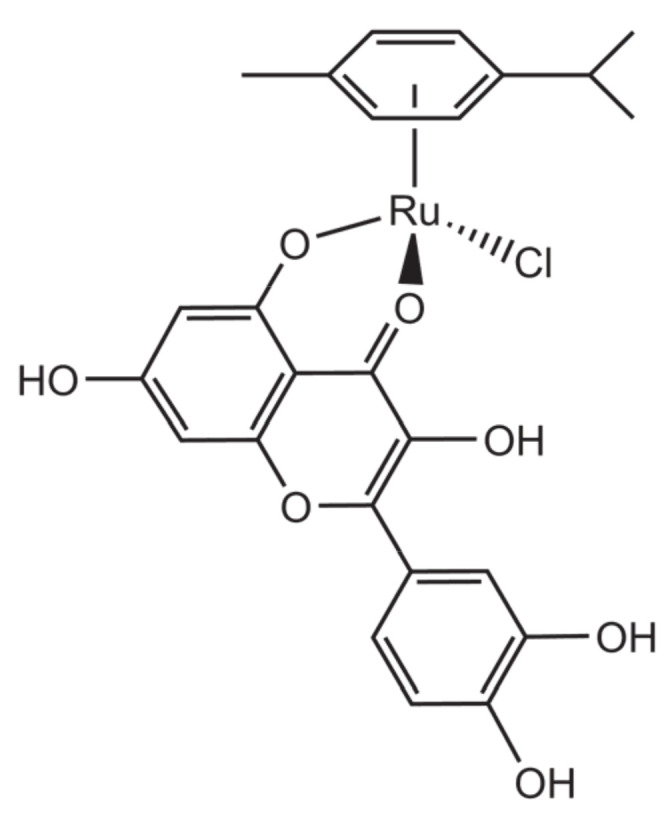
Structures of [Ru(*p*-cym)(chrysin)Cl] (left) and [Ru(*p*-cym)(thiochrysin)Cl] (right), where thiochrysin stands for 5-oxo-7-hydroxy-2-phenyl-4*H*-chromen-4-thionate. The structures are represented using the ball-and-stick style. Redrawn from the atomic coordinates available at the CCDC (refcodes KAYHAW and KAYHEA, respectively) using the software Mercury 3.7.

Thiolation of flavonoids can help improve their biologic activities by making them more hydrophobic. Combining flavonoids and thioflavonoids with a ruthenium scaffold for further stability was the rationale behind the development and evaluation of two complexes as potential anti-thrombotic agents: [Ru(*p*-cym)(chrysin)Cl] and [Ru(*p*-cym)(thiochrysin)Cl] [48]. The structures of the complexes are represented in the Figure 13.

Both complexes had good anti-aggregating activity in washed platelet samples, with 6.25 μM of [Ru(*p*-cym)(chrysin)Cl] inhibiting c.a. 32% aggregation (induced by cross-linked collagen-related peptide and in regard to the control) and [Ru(*p*-cym)(thiochrysin)Cl] inhibiting 60% platelet aggregation; at higher concentrations, e.g., 100 μM, the effect was much stronger, with the complexes inhibiting 90–92% platelet aggregation. The complexes were further demonstrated to interfere with several inter-platelet signalling pathways involved in aggregation, such as the integrin αIIbβ3 inside-out and outside-in signalling paths, the phosphoinositide 3-kinase (PI3K) pathway (involved in calcium mobilisation that leads to platelet activation) and the release of granules (which contribute to recruit other platelets). Moreover, Ru[Ru(*p*-cym)(thiochrysin)Cl] has able to inhibit in vitro thrombus formation and it was shown to affect haemostasis in mice [48].

## 5. Conclusions

In this review we present and discuss the small but already quite diverse collection of ruthenium complexes with flavonoid ligands. Of note, besides the flavonoid, one can find a large variety of ligands completing the ruthenium coordination sphere, from simple solvent molecules such as dimethylsulfoxide and water to a range of hetero/aromatic and macrocyclic ligands that may or may not act as “spectator” ligands. In fact, while their main role is to round up the coordination sphere, their role in the biological activity of the resulting complex is typically not innocent—these ligands help define the polarity of the complex, and therefore its aqueous solubility and permeability, i.e., the ability to cross biological membranes. This is patent in the case of [Ru(bpy)_2_(3-hydroxyflavone)Cl] [42], able to permeate the thick lipopolysaccharide envelope of the Gram-negative bacterium *A. baumannii* and to inactivate it, while the corresponding 1,10-phenanthroline complex, being less polar and less water soluble, had no activity against this bacterium. Another example of contribution towards biological activity is found in complexes with the *p*-cymene ligand, which can participate in interactions with topoisomerase II, an important molecular target for antitumour activity [34].

Most of the reported ruthenoflavonoid complexes are directed at antitumour activity. Research in this field of medicine was triggered by the successful transition of Ru(III) complexes, such as NAMI-A and NKP1339, to clinical trials [49], albeit these did not forego into clinical use. Presently, the interest in antitumor ruthenium-based drugs remains high, being partly fuelled by a Ru(II) polypyridyl complex, TLD1433, which has successfully completed phase I clinical trials [50] and is under phase II trials for the treatment of bladder cancer by photodynamic therapy. In the case of ruthenoflavonoid complexes, the association of a bioactive ligand—the flavonoid itself—is often expected to bring a synergic action with the ruthenium centre, leading with superior activity. This effect is, nevertheless, not straightforward as it was observed for the majority of ruthenium polypyridyl flavonoid complexes [31,32] but not with ruthenium trithiacyclononane flavonoid complexes [33] nor with some ruthenium *p*-cymene flavonoid complexes [34,35,36].

A few studies have demonstrated that, besides antitumour activity, ruthenium polypyridyl flavonoid complexes also exhibit interesting antimicrobial action. While still preliminary, these studies show that antimicrobial applications of ruthenoflavonoids can span a broad range of applications, not only in human infections [31,42] but also in crop-threatening bacteria [43].

Lastly, a new field of application is presented with two examples of ruthenoflavonoid complexes that help prevent and treat cardiovascular diseases, one by reducing cholesterol endogenous production [47] and another by exerting an anti-thrombotic action [48]. While still preliminary, these results open way for a new field of application in which innovative medications are warranted for the growing number of cardiovascular patients emerging in modern societies.

## Figures and Tables

**Table 1:** Cytotoxicity of 3-hydroxyflavone and its ruthenium polypyridyl complexes against colorectal, liver, breast and cervix cancer cell lines. Data of Ru precursors is also shown, for comparison.

Compound	IC_50_ Expressed as Mean ± s.d. (μM)			
	SW620	HepG2	MCF-7	HeLa
3-hydroxyflavone	50.73 ± 22.29	*8.88 ± 17.68*	42.06 ± 21.08	*5.44 ± 31.22*
[Ru(bpy)_2_(3OHflav)][CF_3_SO_3_]	0.75 ± 0.15	2.5 ± 0.67	0.52 ± 0.38	0.78 ± 0.20
[Ru(phen)_2_(3OHflav)][CF_3_SO_3_]	*8.2 ± 46.4*	*11.4 ± 66.0*	8.32 ± 0.86	*19.3 ± 65.9*
[Ru(bpy)_2_Cl_2_]	-	>100	-	>100
[Ru(phen)_2_Cl_2_]	>100	>100	92.38 ± 44.00	>100

Note: Values in italic denote abnormal data distribution.

**Table 2:** Cytotoxicity of flavonoids and their ruthenium *bis*-bathophenanthroline complexes against breast cancer, pharynx carcinoma, glioblastoma, immortalised retinal pigmented epithelium, and immortalised embryonic kidney cell lines.

Compound	IC_50_ Expressed as Mean ± s.d. (μM)					
	MCF-7	FaDU	MDA-MB-435S	U87	RPE-1	HEK293
5-hydroxyflavone	>100	>100	>100	>100	>100	>100
[Ru(bphen)_2_(5OHFlv)][PF_6_]	>50	38.2 ± 5.2	24.5 ± 1.9	30.7 ± 1.5	19.7 ± 8.2	26.5 ± 3.2
genistein	>100	>100	>100	>100	>100	75.9 ± 0.8
[Ru(bphen)_2_(gen)][PF_6_]	16.7 ± 3.9	5.2 ± 0.7	2.6 ± 0.4	5.2 ± 1.7	2.4 ± 0.8	0.7 ± 0.1
chrysin	62.6 ± 3.2	95.1 ± 11.6	79.4 ± 8.1	91.1 ± 13.8	>100	26.8 ± 2.8
[Ru(bphen)_2_(chr)][CF_3_SO_3_]	>50	>50	27.73 ± 5.33	25.59 ± 0.29	23.21 ± 8.08	33.0 ± 3.3
morin	>100	>100	>100	>100	>100	>100
[Ru(bphen)_2_(mor)][CF_3_SO_3_]	>50	>50	>50	>50	>50	>50
[Ru(bphen)_2_Cl_2_]	>50	>50	27.7 ± 5.3	25.6 ± 0.3	3.1 ± 0.3	12.1 ± 1.30
cisplatin	19.7 ± 1.6	5.2 ± 0.2	17.6 ± 0.5	6.9 ± 0.5	39.9 ± 9.1	2.3 ± 0.7
doxorubicin	9.4 ± 1.4	1.6 ± 0.2	5.6 ± 1.4	0.6 ± 0.03	14.9 ± 1.3	0.2 ± 0.03

**Table 3:** Topoisomerase in vitro inhibition and cytotoxicity, expressed by the IC_50_ values in μM (mean ± s.d.) at 96 h of incubation of flavonol derivatives and their corresponding ruthenium *p*-cymene complexes against human ovarian, colon, non-small cell lung, urinary bladder, large cell lung, and pancreatic carcinoma cell lines. Cisplatin is used as the reference drug.

Compound ^1^	Topoisom	CH1	SW480	A549	5637	LCLC-103H	DAN-G	Ref
flavonol	+	1.9 ± 0.2	11 ± 3	25 ± 10	n.d.	n.d.	n.d.	[34]
[Ru(*p*-cym)(flavonol)Cl]	++	2.1 ± 0.2	9.6 ± 1.5	20 ± 2	11 ± 5	13 ± 6	12 ± 2	[34,35]
4′-methylflavonol	+	1.1 ± 0.1	6.3 ± 1.1	81 ± 9	n.d.	n.d.	n.d.	[34]
[Ru(*p*-cym)(4′MeFlv)Cl]	++	1.8 ± 0.2	7.2 ± 0.5	17 ± 2	5.7 ± 3.2	5.2 ± 0.8	6.6 ± 2.5	[34,35]
3′,4′-dimethoxyflavonol	n.d.	2.1 ± 0.2	> 25	> 25	n.d.	n.d.	n.d.	[36]
[Ru(*p*-cym)(3′,4′dMFlv)Cl]	n.d.	2.2 ± 0.5	8.7 ± 0.8	18 ± 2	n.d.	n.d.	n.d.	[36]
3′,5′-dimethoxyflavonol	n.d.	1.4 ± 0.2	> 25	> 25	n.d.	n.d.	n.d.	[36]
[Ru(*p*-cym)(3′,5′dMFlv)Cl]	n.d.	1.5 ± 0.2	4.5 ± 0.2	9.0 ± 0.5	n.d.	n.d.	n.d.	[36]
3′,4′,5′-trimethoxyflavonol	n.d.	2.0 ± 0.2	8.6 ± 1.5	> 25	n.d.	n.d.	n.d.	[36]
[Ru(*p*-cym)(3′,4′,5′tMFlv)Cl]	n.d.	2.5 ± 0.3	9.7 ± 1.9	23 ± 5	n.d.	n.d.	n.d.	[36]
4′-fluoroflavonol	+	1.56 ± 0.04	7.0 ± 0.9	37 ± 10	n.d.	n.d.	n.d.	[34]
[Ru(*p*-cym)(4′FFlv)Cl]	++	1.7 ± 0.4	7.9 ± 2.1	18 ± 1	33 ± 5	5.5 ± 5.2	12 ± 2	[34,35]
[Ru(*p*-cym)(3′FFlv)Cl]	n.d.	1.5 ± 0.1	7.0 ± 1.0	15 ± 1	4.3 ± 2.5	4.3 ± 1.1	5.3 ± 1.6	[35]
[Ru(*p*-cym)(2′FFlv)Cl]	n.d.	4.0 ± 0.8	24 ± 3	30 ± 1	n.d.	n.d.	n.d.	[35]
2′,6′-difluoroflavonol	n.d.	18 ± 1	> 25	> 25	n.d.	n.d.	n.d.	[36]
[Ru(*p*-cym)(2′,6′dFFlv)Cl]	n.d.	5.1 ± 0.8	20 ± 4	55 ± 15	n.d.	n.d.	n.d.	[36]
4′-chloroflavonol	++	0.60 ± 0.10	3.7 ± 0.4	7.9 ± 1.2	n.d.	n.d.	n.d.	[34]
[Ru(*p*-cym)(4′ClFlv)Cl]	+++	0.86 ± 0.06	3.8 ± 0.5	9.5 ± 0.5	3.3 ± 1.1	13 ± 1	19 ± 7	[34,35]
[Ru(*p*-cym)(3′ClFlv)Cl]	n.d.	1.0 ± 0.1	7.0 ± 0.7	12 ± 2	30 ± 2	5.0 ± 3.5	19 ± 5	[35]
[Ru(*p*-cym)(2′ClFlv)Cl]	n.d.	7.9 ± 0.6	26 ± 1	51 ± 5	n.d.	n.d.	n.d.	[35]
cisplatin	-	0.14± 0.03	3.3 ± 0.4	1.3 ± 0.4	n.d.	n.d.	n.d.	[34]

^1^ Abbreviations: 4′MeFlv = 4’-methylflavonol, dMFlv = dimethoxylflavonol, tMFlv = trimethylflavonol, FFlv = fluoroflavonol, dFFlv = difluoroflavonol, ClFlv = chloroflavonol, n.d. = not determined.

**Table 4:** Antimicrobial activity, measured by the diameter of the inhibition zone using the disc diffusion method, of ruthenium flavonoid complexes with 2,2′-bipyridine and phenanthroline ligands, compared to the activities of the ruthenium precursor, *cis*-[Ru(bpy)_2_Cl_2_]. DMSO was used as a negative control and drugs vancomycin, gentamicin and nystatin were used as positive controls.

Compound	*Staphylococcus aureus* ATCC 25923	*Enterococcus faecalis* ATCC 19433	*Streptococcus* β-hemolytic group A	Methicillin-resistant *Staphylococcus aureus*	*Klebsiella pneumoniae* ATCC 1705	*Acinetobacter baumannii* ATCC-BAA 747	*Pseudomonas aeruginosa*	*Escherichia coli*	*Candida albicans*
	Diameter of Inhibition Zone/mm								
quercetin	17	15	–	21	–	18	–	–	–
[Ru(bpy)_2_(quercetin)][CF_3_SO_3_]	–	–	–	–	–	–	–	–	15
[Ru(phen)_2_(quercetin)][CF_3_SO_3_]	–	–	–	–	–	16	–	–	–
morin	–	–	–	–	–	16	–	–	–
[Ru(bpy)_2_(morin)][CF_3_SO_3_]	–	–	–	–	–	14	–	–	–
[Ru(phen)_2_(morin)][CF_3_SO_3_]	–	–	–	–	–	14	–	–	12.5
chrysin	–	–	–	–	–	14	–	–	14
[Ru(bpy)_2_(chrysin)][CF_3_SO_3_]	15	–	15	16	–	13	–	–	17
[Ru(phen)_2_(chrysin)][CF_3_SO_3_]	–	–	–	–	–	17	–	–	–
flavonol	–	–	–	12	–	16.5	–	–	18.5
[Ru(bpy)_2_(flavonol)][CF_3_SO_3_]	25	20	20	26	–	16	13	–	28
[Ru(phen)_2_(flavonol)][CF_3_SO_3_]	–	–	–	14	–	14	–	–	–
*cis*-[Ru(bpy)_2_Cl_2_]	–	–	–	–	–	15	–	–	14
DMSO	–	–	–	–	–	–	–	–	–
vancomycin	27	26	35	–	–	–	–	–	–
gentamicin	–	–	–	20	25	35	36	21	–
nystatin	–	–	–	–	–	–	–	–	28

Notes: Compounds were tested as DMSO solution at a concentration of 2 g·L^−1^ using aliquot with 50 μL volume, which were inserted into wells drilled in the culture medium; the diameter of the inhibition zones were measured after 24 h incubation at 37 °C [31].

## Data Availability

Not applicable.
